# Inhibition of N1-Src kinase by a specific SH3 peptide ligand reveals a role for N1-Src in neurite elongation by L1-CAM

**DOI:** 10.1038/srep43106

**Published:** 2017-02-21

**Authors:** Sarah Keenan, Sarah J. Wetherill, Christopher I. Ugbode, Sangeeta Chawla, William J. Brackenbury, Gareth J. O. Evans

**Affiliations:** 1 Department of Biology, University of York, Wentworth Way, York, YO10 5DD, UK

## Abstract

In the mammalian brain the ubiquitous tyrosine kinase, C-Src, undergoes splicing to insert short sequences in the SH3 domain to yield N1- and N2-Src. We and others have previously shown that the N-Srcs have altered substrate specificity and kinase activity compared to C-Src. However, the exact functions of the N-Srcs are unknown and it is likely that N-Src signalling events have been misattributed to C-Src because they cannot be distinguished by conventional Src inhibitors that target the kinase domain. By screening a peptide phage display library, we discovered a novel ligand (PDN1) that targets the unique SH3 domain of N1-Src and inhibits N1-Src in cells. In cultured neurons, PDN1 fused to a fluorescent protein inhibited neurite outgrowth, an effect that was mimicked by shRNA targeting the N1-Src microexon. PDN1 also inhibited L1-CAM-dependent neurite elongation in cerebellar granule neurons, a pathway previously shown to be disrupted in Src^−/−^ mice. PDN1 therefore represents a novel tool for distinguishing the functions of N1-Src and C-Src in neurons and is a starting point for the development of a small molecule inhibitor of N1-Src.

Basal protein tyrosine phosphorylation is maintained at low levels in cells and is stimulated by signalling pathways that mediate processes such as proliferation, migration and differentiation. In the brain, signalling by non-receptor tyrosine kinases of the Src family (SFK) is upregulated during development, with roles in neuronal differentiation and axon guidance^1^. N1- and N2-Src are neuronal splice variants of C-Src and contain short inserts in their Src homology 3 (SH3) domains^2^,^3^. SH3 domains bind short proline rich peptide motifs^4^,^5^,^6^, which for C-Src comprise peptides in two orientations: Class I (+xφPxxP; x is any amino acid, + is R or K, φ is hydrophobic) and Class II (PxxPφx+)^7^. The SH3 domain has dual roles in the function of Src kinase, firstly via intramolecular interactions to regulate kinase activity and secondly through intermolecular interactions to facilitate enzyme-substrate docking^7^,^8^,^9^,^10^. We and others have previously demonstrated that both functions of the SH3 domain are altered in the N1-Src splice variant. Thus, N1-Src has a higher constitutive kinase activity^11^ thought to be due to weakened intramolecular interactions and canonical C-Src SH3 ligand peptides do not enhance substrate phosphorylation by N1-Src, suggesting reduced docking^12^. C-Src mutations that enhance kinase activity are oncogenic while surprisingly, the high constitutive activity of the N-Srcs is linked with neuronal differentiation^13^,^14^,^15^,^16^,^17^. Maximal N1-Src activity is detected during prenatal and early postnatal development^2^ and clues for the specific functions of N1-Src in nervous system development have come from studies that overexpressed N1-Src in the nervous system of animal models. A transgenic mouse expressing N1-Src in the cerebellum, driven by a Purkinje cell-specific promoter, displayed aberrant Purkinje dendritic morphology in development, which was worsened by a constitutively active mutation^14^. In *Xenopus* retinal ganglion cells, expression of N1-Src mRNA caused mild impairment of axonogenesis, while in a *Xenopus* epithelial cell line N1-Src induced the growth of processes^15^. The effects of N1-Src on process growth suggest conservation with C-Src in an ability to modulate cytoskeletal dynamics albeit with a different morphological phenotype.

To date, no N1-Src specific substrates have been identified *in vivo* to explain its function, however, it is possible that N1-Src substrates in the brain have been mis-assigned to C-Src due to a lack of specific inhibitors or knockdown models that can distinguish the activities of the kinases. Indeed, the majority of Src kinase inhibitors target the ATP binding site of the catalytic domain, which is identical in C- and the N-Srcs. Hence there is a need for selective inhibitors that can dissect the functions of the closely related Src variants in neurons.

We reasoned that a specific N1-Src SH3 domain ligand could act as a kinase inhibitor by competing for SH3 binding to N1-Src substrates. Using phage display, we identified a specific peptide ligand (PDN1) for the N1-Src SH3 domain. The peptide enhanced phosphorylation byN1-Src, but not C-Src, when fused to a Src substrate and inhibited N1-Src activity in cells. N1-Src was implicated in L1-CAM signalling as PDN1 inhibited L1-CAM dependent neurite elongation in cerebellar granule neurons (CGNs). PDN1 will be useful for studying the functions of N1-Src in neurons and could be further developed to produce a soluble small molecule inhibitor of the kinase.

## Results

### A novel SH3 domain binding motif identified by phage display

**Figure 1:**
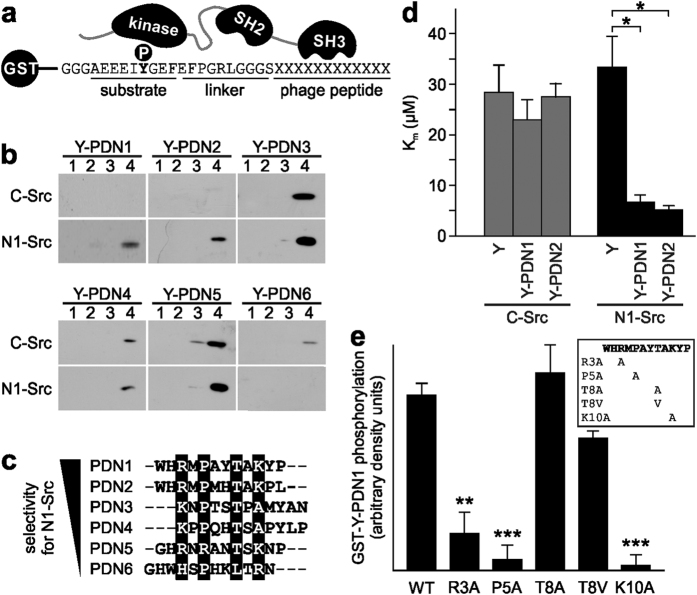
Identification of a novel ligand consensus sequence for the N1-Src SH3 domain. (**a**) Schematic of the kinase assay used to validate the effect of potential SH3 domain ligands on the phosphorylation of an ideal tyrosine motif (Y in bold) located upstream of a 10 aa linker and 12 aa phage peptide. (**b**) GST-Y-PDN1–6 substrates at 0.3, 0.9, 2.8 or 8.3 μM were phosphorylated *in vitro* with 5 nM C- or N1-Src at 30 °C and the phosphorylation detected by anti-phosphotyrosine immunoblot. Immunoblots are representative of three independent experiments. (**c**) Alignment of the phage display peptides ranked by N1-Src selectivity derived from results in (**b**). Shaded residues represent the common consensus sequence. (**d**) Kinetic measurements for GST-Y (Y), GST-Y-PDN1 or GST-Y-PDN2 substrates (see **a** for structure) in the presence of 5 nM C- or N1-Src. Data were plotted as mean K_m_ (μM) ± SEM (n = 3). *P < 0.05 by ANOVA and post-hoc Tukey test. (**e**) GST-Y-PDN1 substrates with the indicated point mutations (see insert) at 8.3 μM were phosphorylated by 5 nM N1-Src and the extent of phosphorylation was measured by densitometry of phosphotyrosine immunoreactivity. Data were plotted as arbitrary units (±SEM, n = 3), normalised to the wild-type (WT) PDN1 peptide. **P < 0.01; ***P < 0.001 by ANOVA and post-hoc Tukey test.

To gain insight into the specificity of the N1-Src SH3 domain we undertook a phage display screen using the GST-N1-Src SH3 domain as bait. This technique was previously used to identify a consensus motif for the C-Src SH3 domain^4^,^18^. The library chosen for panning was a commercially available random 12-mer library containing 2 × 10^9^ unique peptides fused to a coat protein of M13 phage. The panning process was carried out for a total of five rounds and clones were sequenced from round three onwards. Enrichment of certain peptides was observed with 18/18 unique clones for rounds 3 and 4 and only 6/21 unique sequences in round 5, with 17 clones containing the same peptide sequence. These unique peptides were named Phage Display N-Src (PDN) peptides 1–6 (Fig. 1c).

The PDN peptides were tested for specificity for the N1-Src SH3 domain using a kinase assay in which a GST fusion of an ideal Src substrate peptide (Y) and a putative SH3 ligand peptide can distinguish SH3 domain specificity (Fig. 1a). Based on our previous study, we chose a range of substrate concentrations at which an enhancement in phosphorylation by docking to canonical peptide ligands for the C-Src SH3 domain can be detected (8.3 μM and below)^12^. We screened Y-PDN1–6 substrates in kinase assays with C- or N1-Src (Fig. 1b) and found that only Y-PDN1 and Y-PDN2 were specific for N1-Src. Y-PDN3–5 showed little difference in specificity between N1- and C-Src, while Y-PDN6 had a preference for C-Src over N1-Src at the concentrations tested. We then performed a kinetic analysis of Y, Y-PDN1 and Y-PDN2 phosphorylation by C- and N1-Src (Fig. 1c), which revealed that the substrates have K_m_ values (6.5 ± μM and 5 ± μM respectively) for N1-Src, equivalent to that observed for canonical SH3 ligands phosphorylated by C-Src^12^. The corresponding K_m_ values for C-Src phosphorylation of Y-PDN1 and Y-PDN2 were 30 ± μM and 25 ± μM respectively, which were not significantly different to the K_m_ of the Y substrate, indicating no enhancement of substrate phosphorylation by the PDN peptides.

Surprisingly, the sequences of PDN1 (WHRMPAYTAKYP) and PDN2 (WHRMPMHTAKPL) do not contain a PxxP type II helix motif, however, homology alignment of the PDN1–6 sequences in the order of their N1-Src selectivity, revealed potential key residues (Fig. 1d). We therefore undertook alanine substitution mutagenesis of the PDN1 sequence and found that R3, P5 and K10 were essential for the enhancement of substrate phosphorylation (Fig. 1e). Mutation of T8 to alanine or valine had no effect on phosphorylation. Based on the four amino acid differences between PDN1 and PDN2 we were able to eliminate additional residues unlikely to be specificity determinants. We therefore propose a limited consensus for the N1-Src SH3 domain of +xPxxT/A/Vx+. Interestingly, there is a requirement for a positively charged amino acid at both termini of the consensus motif, unlike canonical C-Src Class I and Class II SH3 binding motifs, which have a basic amino acid at either terminus^19^.

### PDN1 inhibits N1-Src *in vitro* and in cells

**Figure 2:**
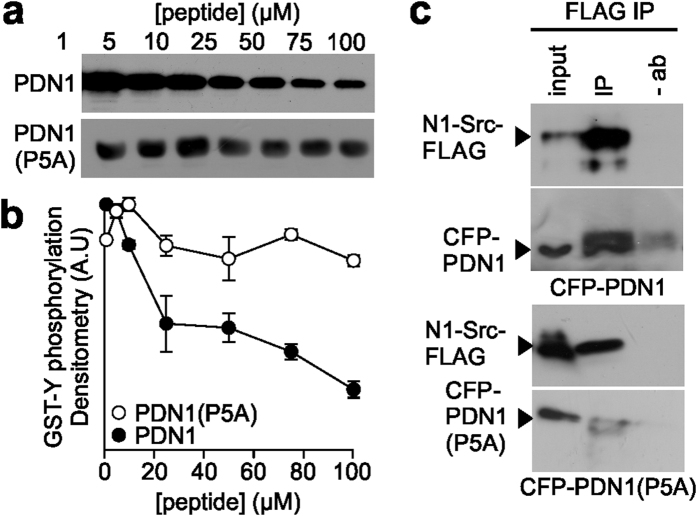
PDN1 inhibits N1-Src activity *in vitro*. GST-Y was incubated with 5 nM N1-Src and the indicated concentrations of either PDN1 or PDN1(P5A) and the extent of phosphorylation determined by densitometry of phosphotyrosine immunoreactivity. Representative immunoblots are shown in (**a**) and in (**b**) the data were plotted as arbitrary density units ± SEM, n = 3. (**c**) COS7 cells co-transfected with N1-Src-FLAG and CFP-PDN1 or CFP-PDN1(P5A) were lysed after 48 h and subject to immunoprecipitation with (IP) or without (−ab) anti-FLAG. The eluates were probed alongside 1% of the input lysate with anti-FLAG (top panels) anti-GFP (bottom panels).

**Figure 3:**
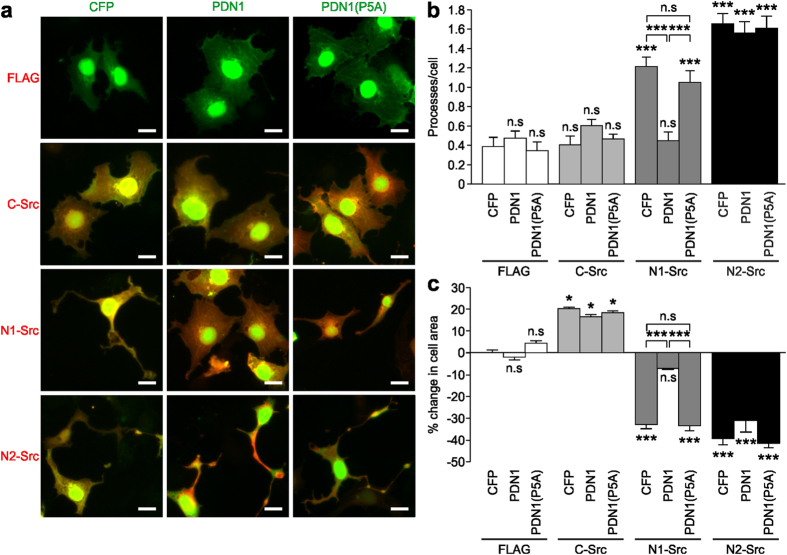
CFP-PDN1 inhibits the biological activity of N1-Src in cells. (**a**) COS7 cells were transfected for 48 h with empty plasmid (FLAG), C-, N1- or N2-Src-FLAG in combination with CFP, CFP-PDN1 or CFP-PDN1(P5A) and processed for immunofluorescence (green = CFP, red = Src-FLAG). Scale bar = 10 μm. Twenty representative fields of view (~50 cells) per condition in each experiment were analysed to quantify (**b**) number of processes per cell and (**c**). Data were plotted as mean ± SEM (n = 3 experiments). Statistical analysis was performed by one way ANOVA and post-hoc Tukey test (*P < 0.05; ***P < 0.001; n.s = not significant).

Previous studies have shown that soluble SH3 ligands can enhance Src family kinase activity *in vitro*^20^,^21^. To test whether free PDN1 activates N1-Src we titrated a soluble free peptide (WHRMPAYTAKYP) into a reaction with N1-Src and the ideal Src substrate GST-Y. Surprisingly, PDN1 inhibited GST-Y phosphorylation in a dose dependent manner (Fig. 2a,b). The PDN1(P5A) mutant caused no significant inhibition consistent with a direct interaction between PDN1 and the SH3 domain of N1-Src. This experiment raised the possibility that PDN1 could act as an N1-Src inhibitor in cells. We first confirmed that PDN1 can bind N1-Src in cells by co-immunoprecipitating N1-Src-FLAG and PDN1 fused to CFP from COS7 cells (Fig. 2c). Co-immunoprecipitation of CFP-PDN1(P5A) with N1-Src-FLAG was greatly reduced. We observed that COS7 cells transfected with N1-Src-FLAG underwent a striking change in morphology within 48 h (Fig. 3a). Similar to that previously observed in *Xenopus* epithelial cells^15^, overexpression of the kinase induced the growth of neurite-like processes (Fig. 3b) and an approximate 35% reduction in area of the cell soma (Fig. 3c). In contrast, and in agreement with previous observations^22^, C-Src did not induce process growth, but induced cell spreading, leading to a 20% increase in cell area (Fig. 3c). Using cell morphology as a readout, we assessed the effect of CFP-PDN1 and PDN1(P5A) on N1-Src activity in cells. Co-transfection of N1-Src with PDN1 prevented both the shrinkage of the cell soma and reduced the number of neurites per cell while the PDN1(P5A) mutant peptide had no significant effect on either parameter (Fig. 3b,c). Furthermore, the peptides had no significant effect upon the morphology of C-Src transfected cells. We also assessed the effect of PDN1 on N2-Src expressing cells because the first five amino acids of its SH3 insert are identical to that in N1-Src^3^. N2-Src induced process outgrowth similar to N1-Src, suggesting overlapping function, but PDN1 did not rescue wild type morphology (Fig. 3b,c), thus the PDN1 peptide is selective for the N1-Src splice variant in cells.

### PDN1 or N1-Src knockdown disrupts neurite outgrowth

**Figure 4:**
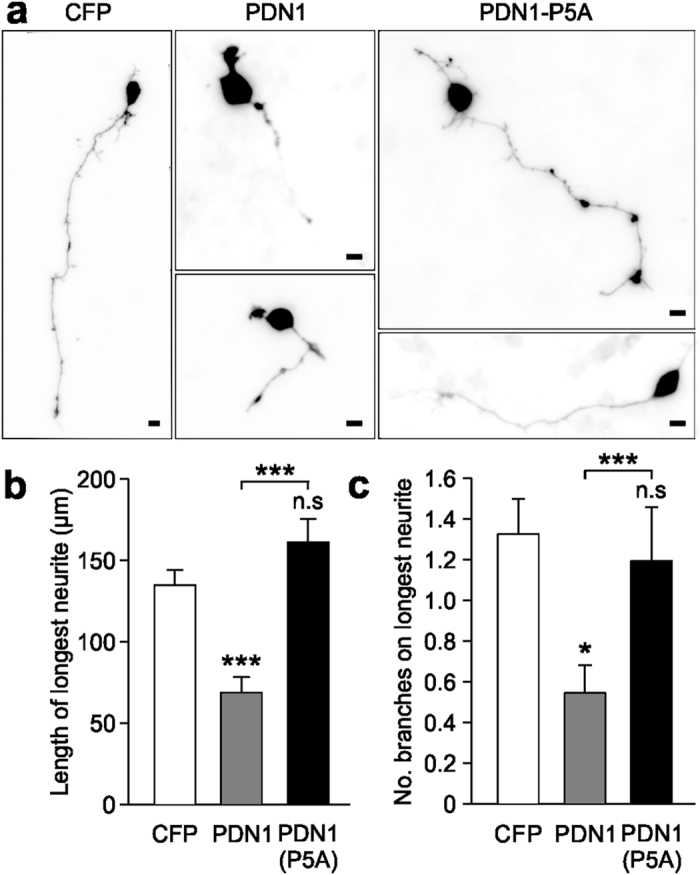
PDN1 inhibits neurite growth in cultured neurons. (**a**) Cultured cerebellar granule neurons were transfected 24 h after plating with CFP, CFP-PDN1 or CFP-PDN1(P5A) for 48 h prior to fixing and processing for immunofluorescence. Scale bar = 10 μm. (**b**,**c**) Length of longest neurite and branches per longest neurite were quantified using the NeuronJ plugin for ImageJ. Data were plotted as mean ± SEM, n = 3 experiments with 30 cells analysed per condition for each experiment. Statistical analysis was performed by one way ANOVA and post-hoc Tukey test (*P < 0.05; ***P < 0.001; n.s = not significant).

**Figure 5:**
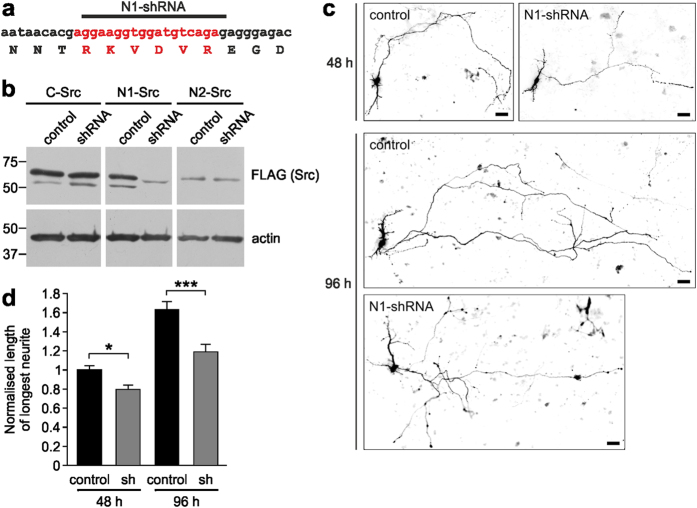
Knockdown of N1-Src phenocopies the action of PDN1 in cultured neurons. (**a**) Schematic of the nucleotide and amino acid sequence of the N1-Src splice insert (red). Black bar indicates the sequence targeted by the N1-Src specific shRNA used in (**b**,**c**,**d**). (**b**) COS7 cells were transfected for 72 h with C-, N1- or N2-Src-FLAG and either pSUPER-GFP (control) or pSUPER-GFP encoding N1-Src specific shRNA. Cell lysates were probed with antibodies to FLAG or actin (loading control). (**c**,**d**) Cultured hippocampal neurons transfected with pSUPER-GFP (control) or pSUPER-GFP encoding N1-Src specific shRNA were analysed for length of longest neurite at 48 or 96 h. Scale bar = 20 μm. Data were normalised to the 48 h control and plotted as mean ± SEM, n = 90 neurons, with 30 cells analysed per condition in 3 independent experiments. Statistical analysis was performed by Kruskal-Wallis and post-hoc Dunn test (*P < 0.05; ***P < 0.001).

We next took advantage of the specificity of PDN1 to investigate the role of endogenous N1-Src in neurite outgrowth in cultured neurons. CFP-PDN1 or -PDN1(P5A) was transfected into 1 DIV CGNs and their morphology analysed after 24 h. During CGN development *in vivo* and immediately after plating *in vitro*, the cells grow two processes before polarising to yield a single axon and the second process retracts to become a dendrite and further dendrites are initiated^23^. The axon then extends further before bifurcating. We observed that the longest neurite, destined to become the axon was reduced in length by PDN1, but not PDN1(P5A) expression (Fig. 4a,b). In addition, longest neurite branching was inhibited in PDN1 transfected neurons (Fig. 4c). To further confirm that N1-Src regulates neurite growth we employed an shRNA expressing plasmid (pSUPER-GFP) to knockdown N1-Src in neurons. By targeting the boundary between Exon 3 and the N1 microexon, an shRNA was designed to be specific for N1-Src (Fig. 5a). Co-expression of the shRNA plasmid with plasmids encoding C-, N1- or N2-Src in fibroblasts confirmed isoform specificity (Fig. 5b). Due to the short N1-Src insert, we were unable to design another suitable shRNA sequence that was specific for N1-Src without also affecting N2-Src expression (data not shown). The successful N1-Src shRNA plasmid was transfected into hippocampal neurons, and the length of the longest neurite in GFP-positive neurons was assessed at 48 h and 96 h post-transfection. The shRNA elicited a significant time-dependent inhibition of neurite growth compared to an empty plasmid (Fig. 5c,d) or a non-targeting shRNA plasmid (Fig. S1). Thus, perturbing N1-Src activity with PDN1 or knockdown of the protein produces the same neurite phenotype in primary neuronal cultures.

### PDN1 perturbs L1-CAM-dependent neurite elongation

**Figure 6:**
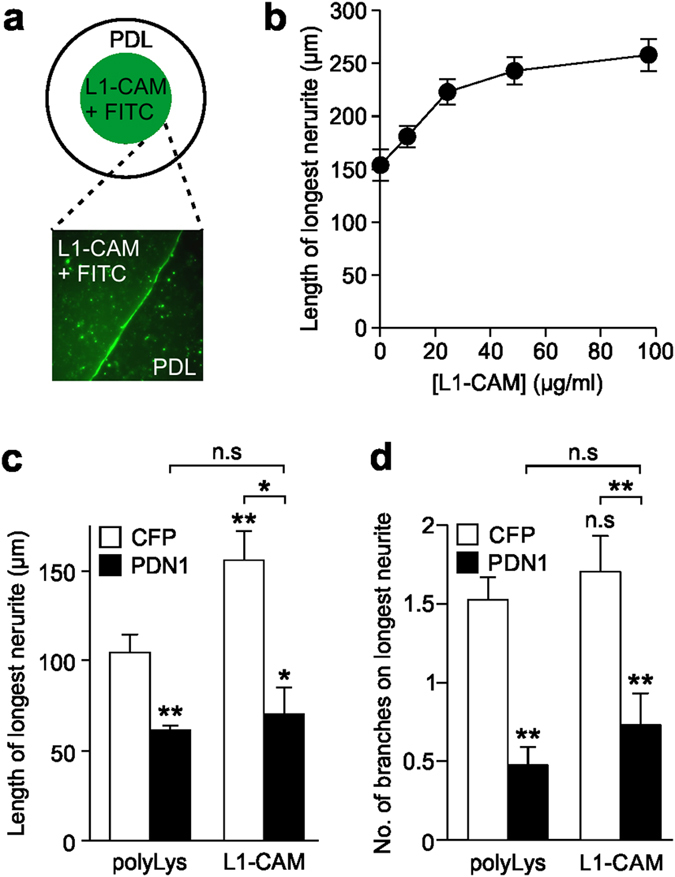
PDN1 inhibits neurite elongation mediated by L1-CAM. (**a**) Schematic of the poly-D-lysine (polyLys) coated coverslips treated with a droplet of L1-CAM and FITC. Inset shows the visible border between FITC/L1-CAM and polyLys coating. (**b**) Cultured cerebellar granule neurons transfected with CFP were grown on the indicated concentrations of L1-CAM and the length of the longest neurite (axon) determined. (**c**,**d**) CGNs were plated on polyLys coated coverslips with a region of 100 μg/ml L1-CAM (see **a**) and transfected after 24 h with CFP (control) or CFP-PDN1 for 48 h prior to fixing and processing for fluorescence microscopy. Length of longest neurite (**c**) and branches on the longest neurite (**d**) were quantified for neurons in the polyLys or L1-CAM regions using the NeuronJ plugin for ImageJ. All data were plotted as mean ± SEM, n = 3 experiments with 30 cells analysed per condition for each experiment. Statistical analysis was performed by one way ANOVA and post-hoc Tukey test (*P < 0.05; **P < 0.01; n.s = not significant).

A previous study of Src^−/−^ mice provided a possible mechanism for the effects of N1-Src upon neurite development. CGNs cultured from Src^−/−^ mice failed to display enhanced neurite growth when plated on coverslips coated with the cell adhesion molecule L1-CAM^24^. Src^−/−^ mice lack C-, N1- and N2-Src, but the isoform responsible for the impaired neurite outgrowth on L1-CAM was not identified in the study. We therefore investigated whether PDN1 affects L1-CAM-dependent neurite growth in cultured CGNs. Recombinant L1-CAM extracellular domain mixed with the fluorescent dye FITC were spotted in the centre of poly-D-lysine (polyLys) coated glass coverslips (Fig. 6a). CGNs were then plated over the whole surface of the coverslip, allowing a direct comparison between neurons growing on L1-CAM and polyLys.

We first demonstrated that L1-CAM increases neurite outgrowth in a dose dependent manner (Fig. 6b). Using a concentration of 25 μg/ml L1-CAM, which induced a 50% increase in neurite length, we then determined the effect of CFP-PDN1 transfection on neurite length on L1-CAM or polyLys. As observed previously, PDN1 inhibited the mean length of the longest neurite grown on polyLys by approximately 40% (Fig. 6c). However, the length of the longest neurite on L1-CAM was not significantly different to that on polyLys, suggesting inhibition of N1-Src by PDN1 is targeting the L1-CAM signalling pathway that mediates neurite elongation.

## Discussion

We addressed the lack of specific reagents for distinguishing the neuronal splice variants of C-Src kinase by developing a specific peptide inhibitor of N1-Src. A 12mer peptide, PDN1, derived from a phage display screen of the N1-Src SH3 domain has shed light on its novel ligand specificity and provided an inhibitor of N1-Src activity *in vitro* and in cells. Furthermore, PDN1 has revealed a role for N1-Src in neurite elongation mediated by L1-CAM signalling.

### A novel SH3 ligand consensus sequence for N1-Src

The PDN1 consensus motif, RxPxxTxK, represents a novel SH3 ligand and lacks a canonical PxxP, thus confirming the six amino acid N1-Src splice insert has a dramatic effect on the structure of its SH3 domain. The ligand binding site in the majority of SH3 domains, including C-Src, comprises three pockets^25^, two of which interact with the core xP motifs and one that adds specificity through binding flanking residues. The fact that PDN1 has critical basic residues at both termini suggests there might be an additional pocket or interface created by the extended n-Src loop. Indeed, one of the few other SH3 domains in nature that contains a substantial insert in the n-Src loop, PI3-kinase^26^, was shown to select a phage display ligand with basic residues at both termini (RSLRPLPPLPPRPPF)^27^ that did not bind the C-Src SH3 domain. The PI-3 kinase n-src loop has three acidic residues that could partake in interactions with ligand flanking residues and thus we predict the aspartate in the N1-insert (underlined in RKVDVR) might co-ordinate an additional basic residue in the consensus ligand. Solving the structure of the N1-Src SH3 domain in complex with PDN1 will reveal the nature of this atypical interaction.

### PDN1 as a N1-Src inhibitor

There are several lines of evidence that support the specific interaction of PDN1 with N1-Src, i) the PDN1 peptide, when fused to an ideal substrate enhanced the specific *in vitro* phosphorylation of an ideal substrate by N1-Src, but not C-Src, ii) PDN1 and N1-Src co-immunoprecipitate from cells, iii) the free soluble PDN1 peptide inhibits the cellular effects of N1-Src and not C- or N2-Src on fibroblast morphology and iv) PDN1 expressed in neurons phenocopies the shRNA mediated knockdown of N1-Src. All of these observations were controlled by a lack of effect by the mutated PDN1(P5A). In contrast to our findings, others have shown activation of SFKs by SH3 ligand peptides *in vitro* with the rationale that the peptide mimics a substrate and promotes the open conformation of the kinase by disrupting binding of the SH3 domain to the SH2-kinase linker^20^,^21^. However, we have previously shown that the N1-Src SH3 domain has a weak affinity for the SH2-kinase linker and is constitutively active in cells^12^. We therefore predict that the overriding action of PDN1 is to compete for SH3 binding to N1-Src substrates and thus prevent their downstream phosphorylation.

### N1-Src functions in neurite elongation

We found that both CFP-PDN1 expression or shRNA knockdown of N1-Src reduces neurite elongation in cultured neurons. We have also observed that overexpression of N1-Src has a similar effect (Fig. S2)^14^,^15^ suggesting that too much or too little N1-Src is detrimental to neurite outgrowth. A potential mechanism for this effect was revealed by the inhibition of L1-CAM-dependent neurite growth by PDN1 in cultured CGNs. CGNs are particularly enriched in N1-Src^11^,^13^,^28^, suggesting it is likely to be the Src splice variant responsible for the loss of L1-CAM-dependent outgrowth in Src^−/−^ CGNs^24^. Src has been shown to phosphorylate the L1-CAM tail at Y1176 to regulate its trafficking^29^ and Src is activated downstream of L1-CAM signalling^30^. Based on our observation that N1-Src overexpression can elicit processes in non-neuronal cells, we favour the idea that N1-Src acts downstream of L1-CAM to drive cytoskeletal rearrangements in neurite outgrowth. Several molecules link L1-CAM to the actin cytoskeleton, such as ezrin^31^ and shootin1^32^, of which ezrin binding to L1-CAM is regulated by Src^33^ and shootin1 acts via cortactin (a Src substrate^34^) to regulate actin flow^35^. Considering that N1-Src has a high constitutive kinase activity in cells^11^,^12^, we postulate that its substrates would be strongly phosphorylated, perhaps providing the driving force for actin remodelling in neurite elongation. In order to identify the specific N1-Src interactors involved in the signalling pathways downstream of L1-CAM, strategies could include N1-Src SH3 pulldowns from developing brain and bioinformatic screening for relevant proteins containing motifs that match the PDN1 consensus sequence.

### Applications for Src inhibition by SH3 ligands

The demonstration that C- and N-Srcs are likely to have distinct functions in the brain highlights the need to develop SH3 domain-specific inhibitors to further study their roles and, where necessary, allow more precise therapeutic inhibition of Src isoforms. For instance, inhibitors that cross the blood brain barrier and target the catalytic domain of Src (such as dasatinib) have been proposed to treat neuronal cancers such as pediatric medulloblastoma^36^,^37^. Inhibition of C-Src could target proliferation, metastasis and angiogenesis in tumours, but the concomitant inhibition of N1-Src could be detrimental to promoting neuronal differentiation of brain cancers such as medulloblastoma and/or cause side effects in the developing pediatric brain. Peptidomimetic ligands of the C-Src SH3 have been developed that potently inhibit SH3 binding to proline rich motifs *in vitro*^38^, but these remain to be tested on the full length kinase or in cells. Finally, N1-Src-specific SH3 domain inhibitors based on PDN1 might be efficacious in other pathological processes in which inhibition of Src signalling in neurons has shown promise, such as ischemic excitotoxicity^39^.

## Materials and Methods

### Materials

Mouse monoclonal anti-phosphotyrosine (clone PY20) was obtained from BD Bioscience (Oxford, UK). Anti-FLAG-M2 mouse monoclonal antibody was from Sigma and rabbit anti-GFP antiserum was a kind gift from Dr. Paul Pryor (University of York). The Ph.D.−12 Phage Display Peptide Library Kit was from New England Biolabs (Hitchin, Herts, UK). Purified L1-CAM was from R&D Systems (Abingdon, UK). Unless stated, all other reagents were from Sigma.

### Plasmids

Plasmids for expressing recombinant ∆80C-Src, N1-Src, C-Src SH3 and N1-Src SH3 in *E. coli* and C-terminal FLAG-tagged full length C- and N1-Src for mammalian expression have been previously described^12^. Plasmids comprising a Src substrate and SH3 ligand fused to GST were prepared by ligating annealed oligonucleotides encoding the ligand peptides (PDN1–6) into a previously described Src substrate plasmid (GST-Y; pGEX6P-1-AEEEIYGEF; ref. 12) with SalI and NotI (see schematic in Fig. 1a). Knockdown of rat N1-Src was achieved using pSUPER.neo-GFP (Oligoengine, Seattle, WA). The N1-Src shRNA was designed to target unique sequences that are not present in C- or N2-Src (5′-GGAAGGTGGATGTCAGAGA-3′). Control experiments were performed with the empty plasmid or a plasmid expressing a non-targeting shRNA (5′-GCGCGATAGCGCTAATAAT-3′).

### Protein purification

Recombinant His- and GST-fusion proteins were expressed and purified according to a previously described protocol^12^.

### Phage display

Phage display screening using the Ph.D−12 Phage Display Peptide Library Kit was performed according to the manufacturer’s instructions. Briefly, for each round of panning, the phage binding step was performed in solution with 1 μM GST-N1-Src SH3 domain and a 100-fold representation of the phage library (2 × 10^11^ plaque forming units; pfu). The SH3-phage complexes were recovered by incubation for 15 min at 4 °C with glutathione agarose (Genscript, Piscataway, NJ). The beads were washed x10 and bound phage were eluted by incubation of the beads in 0.2 M glycine-HCl, pH 2.2, 1 mg/ml BSA for 10 min end-over-end at room temperature and then neutralised with 1 M Tris, pH 9.1. The phage eluate was then amplified and titred in order to calculate the volume required to provide 2 × 10^11^ pfu for the subsequent panning step. Sample clones from titre plates were sequenced from round three and a total of five rounds of panning were performed.

### *In vitro* kinase assays

Phosphorylation reactions were performed as previously described^12^, prepared in kinase reaction buffer (100 mM Tris, 10 mM MgCl_2_, pH 7.5) and initiated by the addition of pre-warmed ATP (0.5 mM final concentration) and incubated at 30 °C for the indicated times. Substrate phosphotyrosine content was determined by immunoblotting with mouse anti-phosphotyrosine (1:1000) and anti-mouse-HRP (1:5000). Densitometric analysis of protein bands on immunoblots was performed with ImageJ. Due to the non-quantitative measurement of V, V_max_ was not determined. K_m_ was calculated from three independent experiments by fitting to Michaelis-Menton equations in the enzyme kinetics module of SigmaPlot (Systat, Chicago, IL).

### Cell culture and transfection

COS7 cells were plated at a density of 3 × 10^4^ cells per well of a 24 well plate or 10^5^ cells per well of a 6 well plate and transfected using EcoTransfect 24 h after plating according to the manufacturer’s instructions (Oz Biosciences, Marseille, France). Rat cerebellar granule and hippocampal neurons were prepared as previously described^40^,^41^ and approved by the Biology Ethics Committee, University of York. The animals were euthanised according to Schedule 1 of the UK Home Office Animals (Scientific Procedures) Act. Neurons were plated at a density of 2.5 × 10^5^ cells per coverslip and transfected after 24 h using a calcium phosphate kit (Promega, Southampton, UK) or Lipofectamine2000 (Invitrogen, Paisley UK) according to the manufacturer’s instructions. Following transfection the medium was replaced with MEM culture medium with 10 μM arabinofuranosyl cytidine for the indicated time before fixing for staining and morphological analysis. For L1-CAM assays, a 10 μl droplet containing 25 μg/ml L1-CAM extracellular domain (R&D Systems) and 1 mg/ml fluorescein isothiocyanate (FITC) was spotted onto the centre of a poly-D-lysine coated coverslip and air dried for 1 h prior to plating neurons.

### Immunocytochemistry

Cells fixed in 4% paraformaldehyde, 4% sucrose were permeabilised in 0.1% Triton,% BSA and stained with primary antibodies (mouse anti-FLAG (M2), 1:1000; rabbit anti-GFP, 1:500) in 1% BSA in PBS for 2 h at room temperature. Secondary antibodies (anti-mouse Alexa Fluor-564 and anti-rabbit Alexa Fluor-488; Invitrogen, Paisley, UK) were applied at 1:500 in 1% BSA in PBS for 1 h in the dark. Images were acquired with a RoleraXR CCD camera (QImaging) on a Nikon TE2000 epifluorescence microscope controlled by SimplePCI software (Hamamatsu).

### Image and statistical analysis

Analysis of COS7 cell area was performed with ImageJ software^42^. The NeuronJ plugin^43^ was used for the quantification of process length and branching in both COS7 cells and neurons. Where appropriate, statistical analyses of the data were performed with SigmaPlot software using one way ANOVA or Kruskal-Wallis tests with post-hoc pairwise comparisons by Tukey or Dunn tests respectively.

## Additional Information

**How to cite this article**: Keenan, S. *et al*. Inhibition of N1-Src kinase by a specific SH3 peptide ligand reveals a role for N1-Src in neurite elongation by L1-CAM. *Sci. Rep.* **7**, 43106; doi: 10.1038/srep43106 (2017).

**Publisher's note:** Springer Nature remains neutral with regard to jurisdictional claims in published maps and institutional affiliations.

## Supplementary Material

Supplementary Information
