# Supplementary material for: Inhibition of N1-Src kinase by a specific SH3 peptide ligand reveals a role for N1-Src in neurite elongation by L1-CAM
Source: Sci Rep. 2017 Feb 21;7:43106. doi: 10.1038/srep43106 (PMC5318895; doi:10.1038/srep43106)
Supplement: Supplementary Information [file srep43106-s1.pdf]

Inhibition of N1-Src kinase by a specific SH3 peptide ligand reveals a role for N1-Src in neurite elongation by L1-CAM

Sarah Keenan, Sarah J. Wetherill, Christopher I. Ugbode, Sangeeta Chawla, William J. Brackenbury and Gareth J.O. Evans[1]

Department of Biology, University of York, Wentworth Way, York, YO10 5DD, UK.

Supplementary Information:

Figures S1 and S2

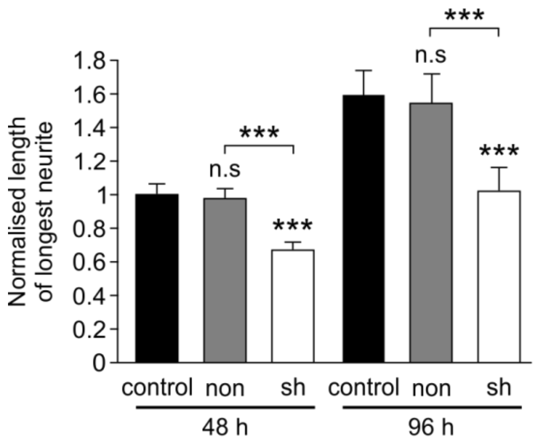

Figure S1. shRNA control plasmids do not affect neurite length. Cultured hippocampal neurons transfected with pSUPER-GFP (control), pSUPER-GFP encoding a non-targeting shRNA (non) or the N1-Src shRNA (sh) were analysed for length of longest neurite at 48 h or 96 h. Data were normalised to the 48 h control and plotted as mean ± SEM, n=50-100 neurons analysed per condition. Statistical analysis was performed by Kruskal-Wallis and post-hoc Dunn test (*** $P<0.001$; n.s, not significant, compared to control at each time point or the indicated comparisons).

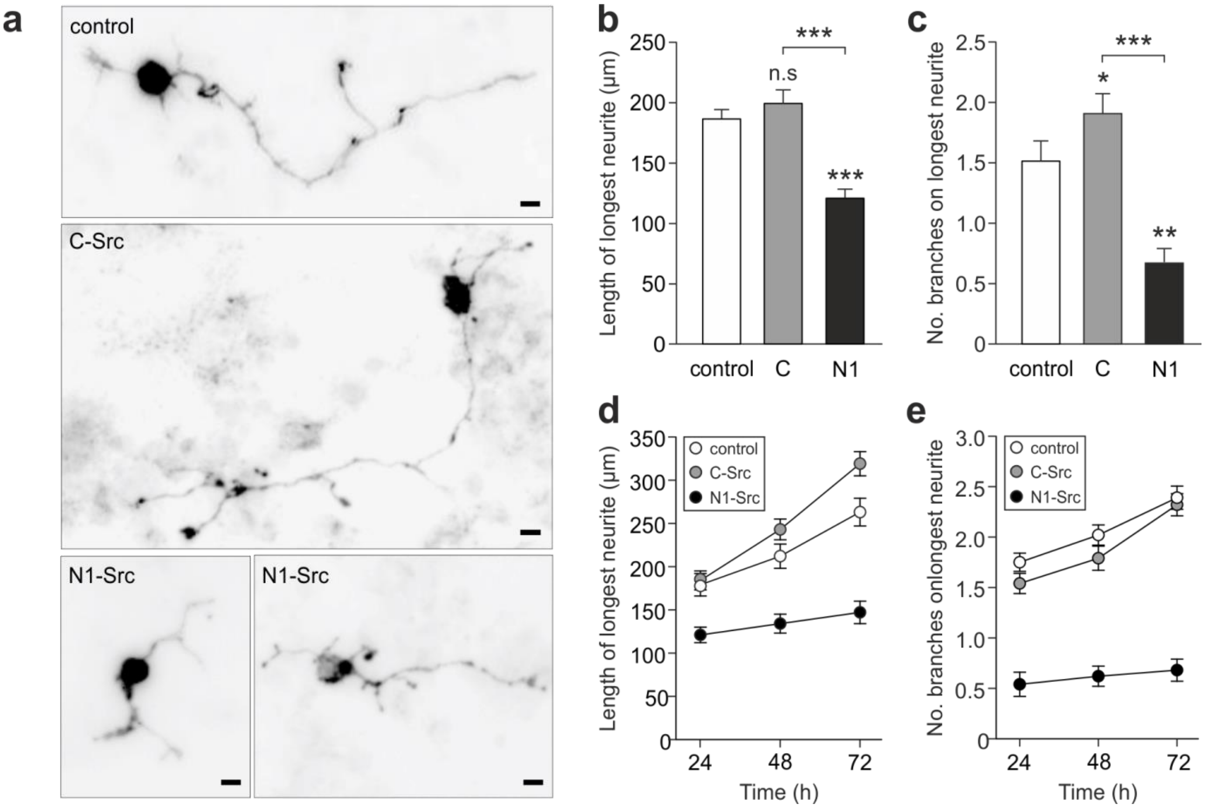

Figure S2. Overexpression of N1-Src in cerebellar granule neurons disrupts neurite outgrowth. A. Twenty four hours after plating, cultured cerebellar granule neurons were transfected with CFP (control), C- or N1-Src-FLAG for 24 (*B* and *C*), 48 or 72 h prior to fixing and processing for immunofluorescence. Scale bar = 10 μm. The NeuronJ plugin for ImageJ was used to quantify the length of the longest neurite (*B*, *D*) and branches on the longest neurite (*C*, *E*). Data were plotted as mean ± SEM, n=3 experiments with 30 cells analysed per condition for each experiment. Statistical analysis was performed by one way ANOVA and post-hoc Tukey test (* $P<0.05$; ** $P<0.01$; *** $P<0.001$).
